# Are Spouses More Likely to be Depressed Than Adult Children in Dementia Care? A Mixed-Methods Study

**DOI:** 10.1093/geroni/igaa057.2126

**Published:** 2020-12-16

**Authors:** Jinyu Liu

**Affiliations:** Weatherhead East Asian institute, New York City, New York, United States

## Abstract

Using a mixed-methods approach, this study examines caregiver burden and depressive symptoms of Chinese American spouses and adult-children who provided care for their spouse or parents with dementia. Quantitative data were collected from a questionnaire-based survey in 124 Chinese caregivers in New York City and narrative data were gathered from in-depth interviews with 27 of these caregivers. The results of linear regression show that there was no difference in objective burden (caring tasks) between spousal and adult-child caregivers, but spousal caregivers reported significantly higher levels of subjective burden and depressive symptoms. Based on the structural equation modeling, it was found that subjective burden significantly mediated the association between being a spousal caregiver on depressive symptoms. The narrative data show that, compared to the adult-child caregivers, spousal caregivers were more likely to express their worries about the sequence of death (what will happen if they die earlier than their care receiver?).

